# Physician- and patient-reported perspectives on myasthenia gravis in Europe: a real-world survey

**DOI:** 10.1186/s13023-023-02727-0

**Published:** 2023-06-29

**Authors:** Milada Mahic, Ali Bozorg, Jonathan DeCourcy, Keisha Golden, Gregor Gibson, Christian Taylor, Anna Scowcroft

**Affiliations:** 1 UCB Pharma, 208 Bath Road, Slough, SL1 3WE UK; 2 UCB Pharma, Morrisville, NC USA; 3 Adelphi Real World, Bollington, UK

**Keywords:** Myasthenia gravis, Real-world data, Diagnosis, Symptoms, HCRU, Disease management, Quality of life

## Abstract

**Background:**

Myasthenia gravis (MG) is a rare, chronic, debilitating, unpredictable, and potentially life-threatening neuromuscular disease. There is a lack of real-world data on disease management that could be used to further understand and address unmet patient needs and burden. We aimed to provide comprehensive real-world insights in the management of MG in five European countries.

**Methods:**

Data were collected using the Adelphi Real World Disease Specific Programme™ in MG, a point-in-time survey of physicians and their patients with MG in France, Germany, Italy, Spain, and the United Kingdom (UK). Physician- and patient-reported clinical data were collected, including demographics, comorbidities, symptoms, disease history, treatments, healthcare resource utilization (HCRU), and quality of life outcomes.

**Results:**

In total, 144 physicians completed 778 patient record forms from March to July 2020 in the UK, and from June to September 2020 in France, Germany, Italy and Spain. Mean patient age at symptom onset was 47.7 years, with a mean time from symptom onset to diagnosis of 332.4 days (10.97 months). At diagnosis, 65.3% of patients were classified as Myasthenia Gravis Foundation of America Class II or above. Mean number of symptoms reported at diagnosis per patient was five, with ocular myasthenia reported in at least 50% of patients. At time of survey completion, the mean number of symptoms reported per patient was five and ocular myasthenia and ptosis were each still present in more than 50% of patients. Acetylcholinesterase inhibitors were the most commonly prescribed chronic treatments in all countries. Of 657 patients treated with chronic treatment at the time of the survey, 62% continued to experience moderate-to-severe symptoms. On average, 3.1 healthcare professionals (HCPs) were involved in patient management, 6.2 consultations were made per patient with any HCP over the last 12 months, and 178 (22.9%) patients were hospitalized in the last 12 months. Overall, HCRU and disease management were similar across all countries.

**Conclusions:**

Our findings demonstrated the high burden of MG despite current treatment options for patients with MG.

## Background

In any therapeutic area, randomized controlled trials are crucial to obtain the highest level of evidence on the efficacy and safety of an intervention. However, in clinical trials the stringent eligibility criteria, requirements for strict adherence to treatment, and regular clinical assessments are often not representative of the real-world patient population. Therefore, real-world studies are important to better understand the true nature of the disease course and patient management [1]. Real-world data can be particularly insightful in rare diseases, where patient populations available for clinical trials are relatively small and additional insights may be required to understand how to best support patients over the course of their disease [2]. In the European Union (EU), a rare disease is defined as one that affects no more than 1 in 2000 people. There are an estimated 30 million people in the EU affected by 6000 to 8000 different rare diseases [3].

Myasthenia gravis (MG) is a rare, chronic, unpredictable, debilitating, and potentially life-threatening neuromuscular disease, characterized by fluctuating chronic muscle weakness and fatigue. MG is caused by pathogenic immunoglobulin G autoantibodies which can inhibit signal transmission at the neuromuscular junction (NMJ) by binding to various proteins including receptors [4–6]. Most patients with MG (80–88%) have pathogenic IgG autoantibodies against the acetylcholine receptor (AChR), while a small minority are autoantibody-positive for muscle-specific kinase (MuSK, < 10%) or low-density lipoprotein receptor-related protein 4 (LRP4, 1%), or are seronegative (10–15%) [6–10]. In AChR autoantibody-positive MG, the development of these pathogenic autoantibodies activates the complement cascade, causing damage to the NMJ and impairment to muscle contraction [11]. In MG, there is a lack of real-world data that could be used to further understand and address unmet patient needs and burden [12]. While there are evidence-based guidelines that outline recommendations for the management of MG [13], there is little evidence about how patients are managed in routine clinical practice, and on patients’ perspectives of living with MG and the effect that the disease has on their quality of life (QoL) [14].

Adelphi Disease Specific Programmes™ (DSPs) are surveys generating data from real-world clinical practice, collecting point-in-time patient demographic data and treatment practices, in addition to resource-use and QoL data, in specific therapy areas across various countries and varying healthcare systems [15]. Here, we used the MG DSP to conduct a point-in-time observational analysis of European physician-reported clinical data and patient-reported QoL data, aiming to provide comprehensive real-world insights into the management of MG in five European countries.

## Methods

### Study design and data source

Data were obtained via the Adelphi MG DSP, a point-in-time survey of MG-treating physicians and their patients, collected in France, Germany, Italy, Spain and the UK. A complete description of the methods of the survey has been previously published and validated [1, 15, 16]. This research obtained ethics approval from the Western Institutional Review Board, sponsor protocol number: AG8768.

### Study population

Physicians were eligible for inclusion if their primary specialty was identified as neurology, geriatrics or primary-care medicine, and they treated at least one patient with a confirmed diagnosis of MG, based on the judgment and diagnostic skills of the respondent physician. No formal patient selection procedures were in place; however, physicians were asked to provide data for a consecutive series of patients to avoid selection bias.

### Study outcomes

Participating physicians completed online patient record forms for each consulting patient after the consultation. Physician-reported patient characteristics including demographics, comorbidities and concomitant medications, clinical profile (reported symptoms and severity of symptoms reported at time of survey completion), most “troublesome” symptoms, symptoms at diagnosis, disease severity by Myasthenia Gravis Foundation of America (MGFA) classification, and disease history were assessed. The term “troublesome” was based on physician interpretation of symptoms experienced by a patient. The maximum possible number of selections for “troublesome” symptoms was five. Symptoms were checked from a pre-selected list and were designated as “mild”, “moderate” or “severe” by physician judgment. “Ocular myasthenia”, “ptosis” and “diplopia” were all separate options during data collection. Ocular myasthenia was defined as “general weakness of the eye muscles”, ptosis was defined as “drooping of one or both eyelids” and diplopia was defined as “blurred or double vision”. Duration of treatment and healthcare resource utilization (HCRU) were also collected from the physician-reported questionnaire. Acute treatment was described in the questionnaire as ‘rescue or acute treatments’, and options were intravenous immunoglobulin (IVIg), subcutaneous immunoglobulin, high-dose steroids, plasmapheresis, and a free text option for ‘other’. Physicians were asked how many times the patient had been hospitalized in the preceding 12 months because of their MG and for each of these occasions (up to a maximum of five), to record whether the patient was admitted through the emergency room (ER) or spent time in the intensive care unit (ICU) at any point during the hospitalization. For collation of QoL and work productivity data, patients for whom the physician had completed a patient record form were invited to complete a pen and paper form themselves, after consultation. Patient-reported work productivity and QoL were assessed by the Work Productivity and Activity Impairment (WPAI) questionnaire, and the 15-item MG QoL scale (MG-QoL-15r) score, EQ-5D-5L and Visual Analog Scale (VAS) scales, respectively.

### Statistical analysis

Data were analysed using UNICOM^®^ Intelligence Reporter version 7.5 (UNICOM Systems, Inc., Mission Hills, CA, USA).

## Results

### Demographics

In total, 778 physician-completed patient record forms were completed by 144 physicians from March to July 2020 in the UK, and from June to September 2020 in France, Germany, Italy and Spain. Physician-reported patient demographics by country are shown in Table 1. The overall mean age of the patients at time of survey completion was 54.0 years and 52.2% of patients were female. Mean time from symptom onset to survey completion was 5.2 years (range: 0.04–61.3 years; n = 597), and mean time from MG diagnosis to survey completion was 4.0 years (n = 698)**.** In all countries, hypertension was the most prevalent comorbidity (24.4%), followed by anxiety (17.4%) and dyslipidemia (14.8%). The proportion of patients taking at least one medication for a comorbid condition, ranged from 75.4% (Germany) to 93.8% (Spain). Mean total number of prescribed and non-prescribed medications taken per person, including for comorbid conditions and over-the-counter or supplementary medication, was 1.7 (n = 736) and ranged between 1.1 (Italy, n = 144) to 2.4 (Germany, n = 100).

**Table 1:** Patient demographics

	France	Germany	Italy	Spain	UK	Total
Enrolled physicians, n	17	33	32	36	26	144
Completed PRQs, n	128	102	152	244	152	778
Completed PRQs per physician, mean (SD, range), n	7.5 (3.48, 1–10)	3.1 (2.10, 1–10)	4.8 (3.78, 1–10)	6.8 (3.46, 1–10)	5.8 (3.93, 1–10)	5.4 (3.66, 1–10)
Age at form completion, mean (SD), years	52.4 (16.69)	57.6 (14.34)	53.5 (15.37)	54.2 (16.74)	52.9 (17.90)	54.0 (16.44)
Age at symptom onset, mean (SD), years	47.9 (15.39, n = 101)	53.3 (14.28, n = 57)	46.9 (16.30, n = 124)	46.9 (16.85, n = 191)	46.7 (18.34, n = 124)	47.7 (16.65, n = 597)
Age at MG diagnosis, mean (SD), years	48.3 (15.71, n = 107)	54.9 (14.24, n = 98)	47.5 (15.82, n = 129)	49.7 (16.75, n = 216)	48.1 (17.87, n = 148)	49.5 (16.47, n = 698)
Time since first symptoms of MG experienced to survey completion, mean (range), years	3.83 (0.71–16.12)	3.55 (0.19–11.16)	6.49 (0.04–41.36)	5.18 (0.11–39.99)	5.60 (0.12–61.3)	5.16 (0.04–61.33)
Sex, %						
Male	41.4	55.9	48.0	50.8	42.8	47.8
Female	58.6	44.1	52.0	49.2	57.2	52.2
BMI						
Overall mean, kg/m^2^	23.9	26.1	24.4	25.0	25.3	24.9
< 18.5, %	3.1	0	2.6	4.1	0.7	2.4
18.5–24.9, %	64.8	42.2	53.3	45.1	50.0	50.5
25–29.9, %	28.1	49.0	38.2	41.4	38.8	39.1
≥ 30, %	3.9	8.8	5.9	9.4	10.5	8.0
Autoantibody status, n (%)						
AChR+	25 (35.7)	4 (8.7)	31 (38.8)	19 (26.8)	28 (27.5)	107 (29.0)
MuSK+	3 (4.3)	0	5 (6.3)	1 (1.4)	0	9 (2.4)
LRP4+	1 (1.4)	0	0	0	0	1 (0.3)
Seronegative	2 (2.9)	1 (2.2)	8 (10.0)	2 (2.8)	2 (2.0)	15 (4.1)
Thymectomy, n (%)	23 (17.97)	27 (26.47)	44 (28.95)	34 (13.93)	34 (22.37)	162 (20.82)
Time since thymectomy to survey completion, mean (SD), weeks	167.2 (186.64, n = 21)	94.2 (60.64, n = 27)	393.1 (441.17, n = 34)	236.4 (287.05, n = 30)	363.8 (625.07, n = 25)	259.9 (391.01, n = 137)
Patients with ≥ 1 comorbidity, n (%)	91 (71.1)	57 (55.9)	102 (67.1)	178 (73.0)	89 (58.6)	517 (66.5)
*Top 5 most reported comorbidities* (%)						
n	91	57	102	178	89	517
1	Hypertension (25.0)	Hypertension (28.4)	Hypertension (20.4)	Hypertension (29.5)	Hypertension (17.1)	Hypertension (24.4)
2	Anxiety (23.4)	Mild liver disease (7.8)	Anxiety (15.8)	Anxiety (25.0)	Depression (12.5)	Anxiety (17.4)
3	Depression (14.1)	Anxiety (5.9)	Dyslipidemia (12.5)	Dyslipidemia (24.2)	Dyslipidemia (11.2)	Dyslipidemia (14.8)
4	Dyslipidemia (12.5)	Depression (4.9)	Depression (10.5)	Depression (22.1)	Diabetes without chronic complications (9.9)	Depression (14.4)
5	Hashimoto’s thyroiditis (8.6)	Dyslipidemia (3.9)	Diabetes without chronic complications (9.9)	Diabetes without chronic complications (16.0)	Anxiety (9.2)	Diabetes without chronic complications (10.4)
Patients with ≥ 1 comorbid condition taking co-medications, %	83.5 (n = 91)	75.4 (n = 57)	86.3 (n = 102)	93.8 (n = 178)	78.7 (n = 89)	85.9 (n = 517)
Number of co-medications per patient with ≥ 1 comorbid condition, mean	1.6 (n = 91)	2.4 (n = 57)	1.1 (n = 102)	1.6 (n = 178)	2.1 (n = 89)	1.7 (n = 517)
*Top 5 most reported co-medications in patients with *≥ *1 comorbid condition* (%)						
n	91	57	102	178	89	517
1	Other psychological medication (21)	None (25)	Antidepressants (28)	Antidepressants (38)	Statins (26)	Antidepressants (27.1)
2	Antidepressants (21)	ACE inhibitors (23)	Statins (25)	Statins (37)	Antidepressants (24)	Statins (26.7)
3	Statins (19)	Other anti-hypertensive medication (18)	ACE inhibitors (25)	Insulin (23)	None (21)	ACE inhibitors (18.0)
4	None (16)	Beta blockers (14)	Insulin (15)	Non-insulin anti-diabetics (22)	Non-insulin anti-diabetics (19)	Insulin (16.4)
5	Thyroid medication (14)	Statins (12)	None (14)	Diuretics (21)	Insulin (19)	Non-insulin anti-diabetics (15.3)

The denominator equals the number of completed PRQs, unless otherwise specified

*ACE* angiotensin-converting enzyme; *AChR* acetylcholine receptor; *BMI* body mass index; *LRP4* low-density lipoprotein receptor-related protein 4; *MG* myasthenia gravis; *MuSK* muscle-specific kinase; *PRQ* physician-reported questionnaire; *SD* standard deviation

### Clinical profile

Overall, mean age at symptom onset was 47.7 years (n = 597), with a mean time from symptom onset to diagnosis of 10.97 months (332.4 days, n = 575), and mean time from first symptom-related consultation with any physician to diagnosis of 6.92 months (209.7 days, n = 594) (Table 1). In total, 20.8% patients had a thymectomy since their MG diagnosis (Table 1); 51.9% (n = 84/162) of these patients experienced an improvement in disease symptoms and had not yet relapsed (at the time of survey completion), while 29.6% (n = 48/162) patients improved but had relapsed, 15.4% (n = 25/162) did not see any improvement, and 2.5% (n = 4/162) deteriorated further. Prior to diagnosis, 0.9% of patients had a thymectomy. Diagnosing physicians were neurologists for most patients (80.8%, n = 778), but this varied between countries: Germany, 61.8%; France, 78.1%; Spain, 78.3%; Italy, 88.2%; UK, 98.2%. Notably, almost 40% of patients in Germany, and almost 20% of patients in Spain and France were diagnosed by a non-neurologist specialist, including general practitioners, ophthalmologists, internists and geriatricians. Almost one-quarter of patients (24.3%) across all countries had initially been misdiagnosed with another condition. Chronic fatigue syndrome was the most common misdiagnosis in four out of five countries, accounting for 32.8% of misdiagnoses overall (Table 2).

**Table 2:** Proportion of patients initially misdiagnosed and common misdiagnoses

		France (n = 128)	Germany (n = 102)	Italy (n = 152)	Spain (n = 244)	UK (n = 152)	Total (N = 778)
Patients who were misdiagnosed with another condition, n (%)		31 (24.2)	27 (26.5)	26 (17.1)	66 (27.0)	39 (25.7)	189 (24.3)
Top 5 common misdiagnoses in patients who were initially misdiagnosed with another condition (%)	n	31	27	26	66	39	189
	1	Chronic fatigue syndrome (41.9)	Amyotrophic lateral sclerosis (29.6)	Chronic fatigue syndrome (26.9)	Chronic fatigue syndrome (28.8)	Chronic fatigue syndrome (43.6)	Chronic fatigue syndrome (32.8)
	2	Posterior circulation stroke (12.9)	Chronic fatigue syndrome (22.2)	Other (19.2)	Blepharospasm (16.7)	Transient ischemic attack (15.4)	Posterior circulation stroke (8.5)
	3	Multiple sclerosis (12.9)	Botulism (22.2)	Transient ischemic attack (11.5)	Hysteria (12.1)	Posterior circulation stroke (15.4)	Other (6.9)
	4	Critical neuropathy/myopathy (9.7)	Lambert-Eaton myasthenic syndrome (14.8)	Hysteria (11.5)	Critical neuropathy/myopathy (10.6)	Oculopharyngeal muscular dystrophy (10.3)	Multiple sclerosis (6.9)
	5	Chronic inflammatory demyelinating polyneuropathy (9.7)	Heavy metal poisoning (14.8)	Chronic inflammatory demyelinating polyneuropathy (7.7)	Connective tissue disease (10.6)	Multiple sclerosis (5.1)	Transient ischemic attack (6.9)

**Fig. 1:**
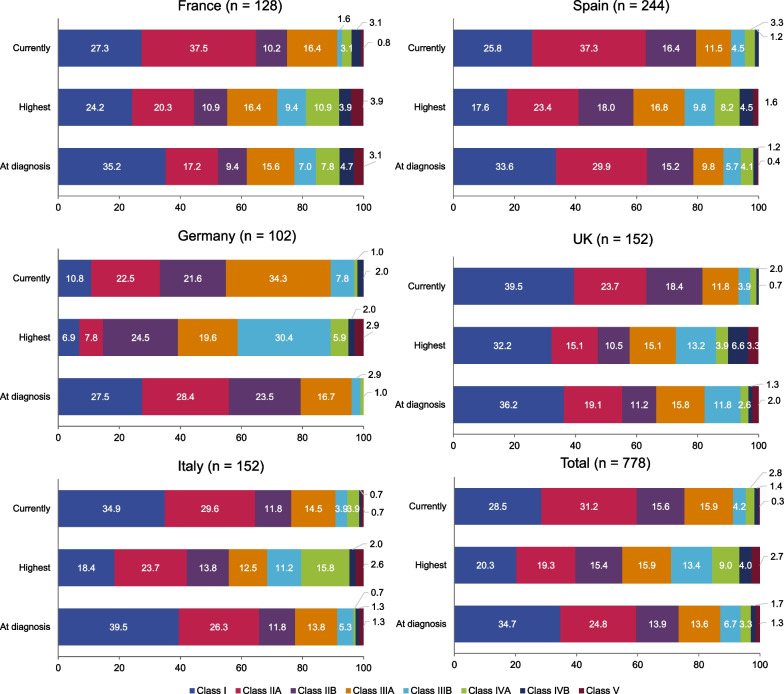
Proportion of patients with each MGFA classification at diagnosis, highest, and currently*. *Current at time of survey completion. MGFA, Myasthenia Gravis Foundation of America

At diagnosis, 65.3% of patients had more than just ocular symptoms (MGFA II–V) and, at time of survey completion, this proportion of patients with generalized MG increased to 71.5% (Fig. 1). From a confirmed MG diagnosis until the time of survey, 45.0% of patients were classified with MGFA Class III or above, meaning they had experienced moderate-to-severe weakness of limb, axial, oropharyngeal and/or respiratory muscles. The five most frequently reported and most troublesome symptoms are presented in Table 3. In general, the mean number of symptoms per patient, and type of symptom, was similar at diagnosis to the time of survey completion. Ocular myasthenia was the most frequently reported symptom at the time of survey completion (60.7%), while general fatigue was reported as being the most “troublesome” symptom overall and in each country. Overall, 13.8% of general fatigue cases were reported as severe, ranging from 5.6% (UK) to 16.8% (Spain). While frequently reported, most cases of ocular myasthenia were mild, and it was generally not regarded as “troublesome”.

**Table 3:** Symptoms at diagnosis and current (at time of survey completion) symptoms, most reported symptoms and most “troublesome” symptoms

		France (n = 128)			Germany (n = 102)			Italy (n = 152)			Spain (n = 244)			UK (n = 152)			Total (N = 778)		
Mean number of symptoms per patient at diagnosis		5			5			5			6			4			5		
Top 5 most reported symptoms at diagnosis		Symptom	n (%)		Symptom	n (%)		Symptom	n (%)		Symptom	n (%)		Symptom	n (%)		Symptom	n (%)	
	1	Ptosis	77 (60.2)		Ocular myasthenia	79 (77.5)		Ocular myasthenia	117 (77.0)		Ptosis	171 (70.1)		Ptosis	92 (60.5)		Ocular myasthenia	525 (67.5)	
	2	General fatigue	69 (53.9)		Muscle ache following physical activity	67 (65.7)		Ptosis	106 (69.7)		Ocular myasthenia	168 (68.9)		Ocular myasthenia	92 (60.5)		Ptosis	501 (64.4)	
	3	Ocular myasthenia	69 (53.9)		Ptosis	55 (53.9)		Diplopia	88 (57.9)		﻿Diplopia	130 (53.3)		﻿Diplopia	70 (46.1)		﻿Diplopia	393 (50.5)	
	4	Diplopia	58 (45.3)		General fatigue	47 (46.1)		General fatigue	60 (39.5)		General fatigue	124 (50.8)		General fatigue	67 (44.1)		General fatigue	367 (47.2)	
	5	Weakness in the arms	57 (44.5)		Diplopia	47 (46.1)		Weakness in the arms	52 (34.2)		Weakness in the arms	99 (40.6)		Weakness in the arms	54 (35.5)		Weakness in the arms	277 (35.6)	
Mean number of current* symptoms per patient		4			7			5			6			3			5		
Top 5 most reported current* symptoms and severity		Symptom	n (%)	Severity, %^†^	Symptom	n (%)	Severity, %^†^	Symptom	n (%)	Severity, %^†^	Symptom	n (%)	Severity, %^†^	Symptom	n (%)	Severity, %^†^	Symptom	n (%)	Severity, %^†^
	1	General fatigue	64 (50.0)	Mild: 32.8 Moderate: 51.6 Severe: 15.6	Ocular myasthenia	78 (76.5)	Mild: 21.8 Moderate: 78.2 Severe: 0.0	Ocular myasthenia	105 (69.1)	Mild: 53.3 Moderate: 45.7 Severe: 1.0	Ptosis	160 (65.6)	Mild: 57.5 Moderate: 36.9 Severe: 5.6	General fatigue	71 (46.7)	Mild: 43.7 Moderate: 50.7 Severe: 5.6	Ocular myasthenia	472 (60.7)	Mild: 54.9 Moderate: 42.4 Severe: 2.8
	2	Ocular myasthenia	62 (48.4)	Mild: 67.7 Moderate: 25.8 Severe: 6.5	Muscle ache following physical activity	71 (69.6)	Mild: 5.6 Moderate: 77.5 Severe: 16.9	Ptosis	84 (55.3)	Mild: 52.4 Moderate: 44.0 Severe: 3.6	Ocular myasthenia	156 (63.9)	Mild: 57.1 Moderate: 39.1 Severe: 3.8	Ocular myasthenia	71 (46.7)	Mild: 77.5 Moderate: 19.7 Severe: 5.6	Ptosis	435 (55.9)	Mild: 55.2 Moderate: 40.0 Severe: 4.8
	3	Weakness in the arms	60 (46.9)	Mild: 38.3 Moderate: 55.0 Severe: 6.7	General fatigue	71 (69.6)	Mild: 18.3 Moderate: 69.0 Severe:12.7	﻿Diplopia	62 (40.8)	Mild: 54.8 Moderate: 45.2 Severe: 0.0	General fatigue	131 (53.7)	Mild: 33.6 Moderate: 49.6 Severe: 16.8	Ptosis	65 (42.8)	Mild: 66.2 Moderate: 27.7 Severe 6.2	General fatigue	398 (51.2)	Mild: 32.4 Moderate: 53.8 Severe: 13.8
	4	Ptosis	58 (45.3)	Mild: 51.7 Moderate: 41.4 Severe: 6.9	Ptosis	68 (66.7)	Mild: 45.6 Moderate: 52.9 Severe: 1.5	General fatigue	61 (40.1)	Mild: 32.8 Moderate: 50.8 Severe: 16.4	﻿Diplopia	105 (43.0)	Mild: 58.1 Moderate: 36.2 Severe: 5.7	﻿Diplopia	52 (34.2)	Mild: 71.2 Moderate: 28.8 Severe: 0.0	﻿Diplopia	298 (38.3)	Mild: 59.4 Moderate: 37.6 Severe: 3.0
	5	Weakness in the legs	48 (37.5)	Mild: 45.8 Moderate: 43.8 Severe: 10.4	Weakness in the neck	63 (61.8)	Mild: 36.5 Moderate: 55.6 Severe: 7.9	Weakness in the legs	45 (29.6)	Mild: 37.8 Moderate: 40.0 Severe: 22.2	Weakness in the arms	104 (42.6)	Mild: 41.3 Moderate: 51.9 Severe: 6.7	Weakness in the arms	47 (30.9)	Mild: 63.8 Moderate: 19.7 Severe: 2.8	Weakness in the arms	291 (37.4)	Mild: 44.0 Moderate: 48.5 Severe: 7.6
﻿Top 5 most “troublesome” symptoms (%)	n	118			96			141			233			141			729		
	1	General fatigue (34.7)			General fatigue (29.2)			General fatigue (27.0)			General fatigue (34.3)			General fatigue (35.5)			General fatigue (32.5)		
	2	Weakness in the arms (24.6)			Muscle ache following physical activity (28.1)			Ocular myasthenia (23.4)			Ptosis (25.3)			Ptosis (22.7)			Ptosis (22.8)		
	3	Weakness in the legs (19.5)			Ptosis (28.1)			Diplopia (22.0)			Diplopia (22.3)			Diplopia (15.6)			Diplopia (19.8)		
	4	Diplopia (17.8)			Weakness in the neck (27.1)			Ptosis (20.6)			Ocular myasthenia (19.3)			Ocular myasthenia (14.2)			Ocular myasthenia (19.6)		
	5	Ocular myasthenia (16.9)			Ocular myasthenia (26.0)			Difficulty swallowing/choking on food (9.9)			Weakness in the legs (16.7)			Weakness in the arms (9.9)			Weakness in the arms (12.9)		

Symptoms were checked from a pre-selected list. “Ocular myasthenia”, “ptosis” and “diplopia” were all separate options during data collection. Ocular myasthenia was defined as “general weakness of the eye muscles”, ptosis was defined as “drooping of one or both eyelids” and diplopia was defined as “blurred or double vision”. “Myasthenic crisis” was not an option in the preselected list

*Current at time of survey completion

^†^Denominator is the number of patients experiencing that symptom

### Treatment

**Fig. 2:**
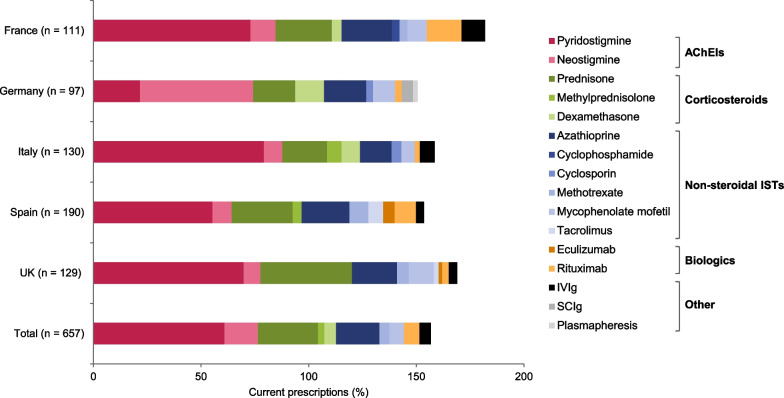
Top 10 most prescribed chronic treatments (current*) in each country. *Current at time of survey completion. Total prescriptions are above 100% as more than one treatment could be selected per patient. IVIg, intravenous immunoglobulin; SCIg, subcutaneous immunoglobulin

Most patients with MG had been prescribed treatment at some point over the course of their disease; only 4.0% of all patients had never received any prescribed treatment for their MG (Table 4). Among patients who were classified as MGFA Class III and above at time of survey completion (n = 192), six (3.1%) patients had never received any prescribed treatment. Acetylcholinesterase inhibitors (AChEIs) were the most commonly prescribed chronic treatments in all countries, followed by prednisone and azathioprine (Fig. 2).

**Table 4:** Patients receiving acute treatment, most frequently prescribed current (at time of survey completion) acute treatments, and reasons for acute treatment

	France (n = 128)	Germany (n = 102)	Italy (n = 152)	Spain (n = 244)	UK (n = 152)	Total (N = 778)
Untreated^†^, n (%)	4 (3.1)	2 (2.0)	7 (4.6)	14 (5.7)	4 (2.6)	31 (4.0)
Previous acute treatment, n (%)	57 (44.5)	27 (26.5)	54 (35.5)	109 (44.7)	51 (33.5)	298 (38.3)
Current* acute treatment	n = 9	n = 3	n = 8	n = 21	n = 6	n = 47
IVIg, n (%)	5 (55.6)	3 (100.0)	7 (87.5)	7 (33.3)	2 (33.3)	24 (51.1)
High dose steroids, n (%)	5 (55.6)	–	–	10 (47.6)	2 (33.3)	17 (36.2)
Plasmapheresis, n (%)	–	–	1 (12.5)	1 (4.8)	–	2 (4.3)
SCIg, n (%)	–	–	–	3 (14.3)	–	3 (6.4)
Other, n (%)	1 (11.1)	–	–	1 (4.8)	2 (33.3)	4 (8.5)
Reasons for acute treatment	n = 9	n = 3	n = 8	n = 21	n = 6	n = 47
Exacerbation^‡^, n (%)	5 (56.6)	2 (66.7)	3 (37.5)	11 (52.4)	3 (50.0)	24 (51.1)
Myasthenic crisis^§^, n (%)	3 (33.3)	1 (33.0)	3 (37.5)	4 (19.0)	1 (16.7)	12 (25.5)
Prior to thymectomy surgery, n (%)	2 (22.2)	–	–	2 (9.5)	–	4 (8.5)
Patient not responding to maintenance/chronic therapy, n (%)	1 (11.1)	–	1 (12.5)	1 (4.8)	–	3 (6.4)
Prior to an unrelated surgery, n (%)	–	–	1 (12.5)	1 (4.8)	–	2 (4.6)
Patient needs fast onset of action until maintenance therapy, n (%)	–	–	–	2 (9.5)	2 (33.3)	4 (8.5)
Don’t know, n (%)	–	–	–	1 (4.8)	–	1 (2.1)

Data are not adjusted

*IVIg* intravenous immunoglobulin; *SCIg* subcutaneous immunoglobulin

*Current at time of survey completion

^†^Patients who have never received any prescribed treatment

^‡^Physician-defined, in response to: “Patient suffering from exacerbated symptoms/relapse (but not a crisis)”

^§^Physician-defined, in response to: “Patient was in myasthenic crisis”

However, despite treatment, some patients still experienced moderate or severe symptoms at time of survey completion: 657 patients were receiving chronic treatment, of whom 410 (62.4%) still experienced moderate or severe symptoms. Of patients who still experienced moderate or severe symptoms, 219 patients had only received first-line chronic treatment (AChEI and/or steroids) and of those, 70 patients had received acute treatment. Forty-six patients who were still experiencing moderate to severe symptoms were being treated with second-line non-steroidal immunosuppressants as part of their chronic treatment regimen.

The total number of patients who were receiving or had previously received acute treatment is reported in Table 4. Overall, 298 (38.3%) patients had received acute treatment, and IVIg was the most prescribed acute treatment in all countries except Spain (high-dose steroids). The most common reasons for prescribing acute treatment in all countries were exacerbations or myasthenic crises, as judged by the physician (Table 4).

### HCRU

**Fig. 3:**
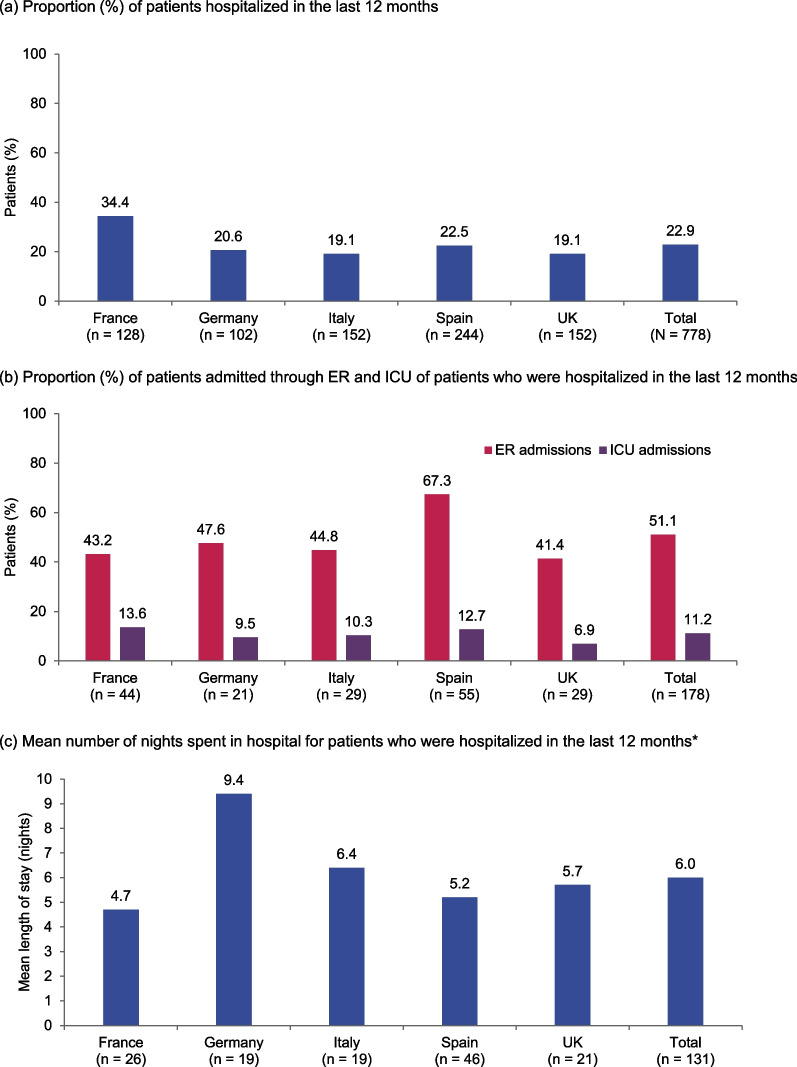
Physician-defined hospital admissions and length of stay in the last 12 months. **a** Proportion (%) of patients hospitalized in the last 12 months. **b** Proportion (%) of patients admitted through ER and ICU of patients who were hospitalized in the last 12 months. **c** Mean number of nights spent in hospital for patients who were hospitalized in the last 12 months*. Data are based on answers to the following questions from the physician-reported questionnaire: **a** “How many times has the patient been hospitalized because of their MG?”; **b** “Was the patient in ICU/ER at any point during the hospitalization?”; and **c** “What was the time spent in hospital?”. *Excludes day cases and “Don’t know” answers. ER, emergency room; ICU, intensive care unit

On average, 3.1 healthcare professionals (HCPs) were involved in patient management, with the top three overall being neurologists (92.5%), primary care physicians (78.3%), and ophthalmologists (17.7%) (Table 5). Overall, each patient had an average of 6.2 consultations with HCPs over the last 12 months. Of patients who were untreated (n = 31), primary care physicians (n = 16, 51.6%) and neurologists (n = 8, 25.8%) were the HCPs most involved in their overall management. Approximately a quarter of the patients in each country were hospitalized in the last 12 months, with half of these admitted via the ER and 11.2% of patients admitted to the ICU (Fig. 3a, b). Of those who stayed overnight (n = 131, 74% of hospitalized patients in the last 12 months), the mean length of stay was 6 nights (Fig. 3c). The most common reason for admission was to receive IVIg (31.5%), followed by treating a complication (24.7%).

**Table 5:** HCP involvement in patient management and consultations in the last 12 months

	France (n = 128)	Germany (n = 102)	Italy (n = 152)	Spain (n = 244)	UK (n = 152)	Total (N = 778)
Most frequently involved HCPs in patient management*						
Primary care physician, %	75.8	87.3	60.5	85.2	80.9	78.3
Neurologist, %	89.8	79.4	96.1	93.9	98.0	92.5
Ophthalmologist, %	25.8	28.4	–	–	–	17.7
Pulmonologist, %	–	–	18.4	–	–	–
Internist, %	–	–	–	23.4	–	–
Neuromuscular specialist nurse, %	–	–	–	–	15.1	–
Total number of HCPs involved in patient management, mean	2.8	3.5	2.7	3.3	2.8	3.1
Number of consultations with HCPs in last 12 months, mean	6.3	9.8	4.3	7.2	3.8	6.2

*HCPs* healthcare professionals

*The top three most frequently involved HCPs in patient management are reported for each country

### Patient-reported work productivity and QoL

**Fig. 4:**
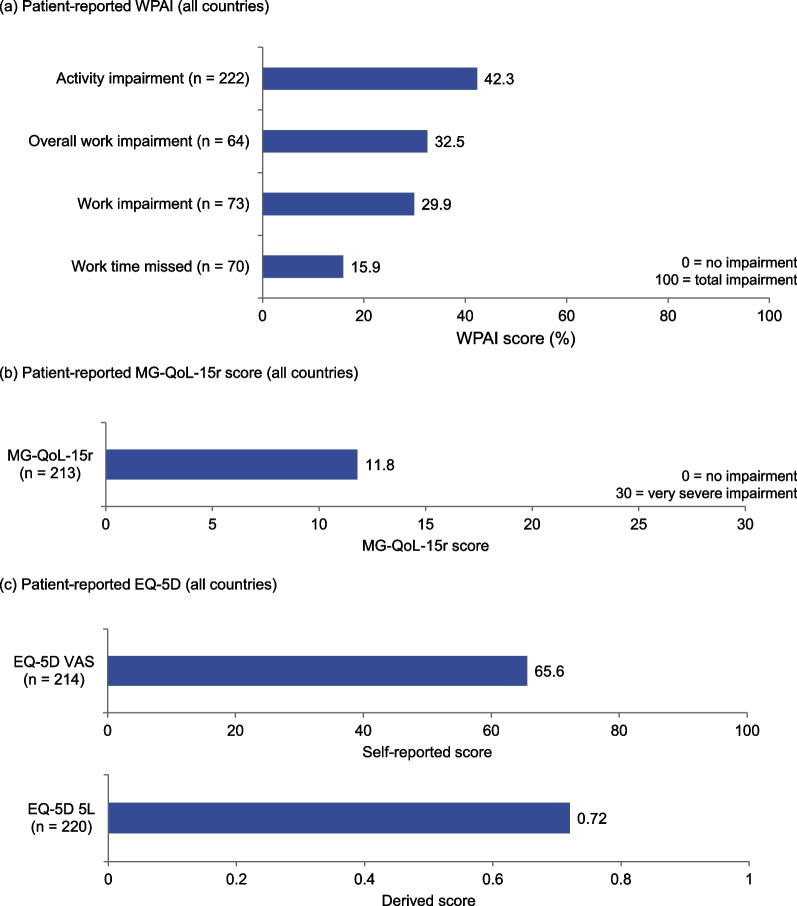
Patient-reported work productivity and QoL. **a** Patient-reported WPAI (all countries). **b** Patient-reported MG-QoL-15r score (all countries). **c** Patient-reported EQ-5D (all countries). MG-QoL-15r, 15-item Myasthenia Gravis Quality of Life scale; VAS, Visual Analog Scale; WPAI, Work Productivity and Activity Impairment

In total, 226 patient-completed forms and 28 caregiver-completed forms were received. WPAI and EQ-5D scores from the patient-completed forms are presented in Fig. 4 for all countries combined. Mean WPAI scores for patient-reported work time missed, work impairment and activity impairment were between 16 and 42% (Fig. 4a), and patients reported an overall MG-QoL-15r score of 11.8 (standard deviation = 7.43) out of 30 (n = 213), where 0 represents no impairment and 30 represents very severe impairment (Fig. 4b). EQ-5D VAS and EQ-5D-5L scores were 65.6 out of 100 (n = 214) and 0.72 out of 1.00 (n = 220) respectively (Fig. 4c), where 0 represents the worst health state on both scales. Compared with a mean VAS for the general population of France, Germany, Italy, Spain and the UK combined of 77.8 [17], these data suggest that MG had a moderate reduction on patients’ QoL.

## Discussion

These data provide a comprehensive set of real-world insights into the management of MG from five European countries and confirm previous findings from the United States that a significant number of people living with MG have chronically uncontrolled disease [18].

The proportion of patients who had more than just ocular symptoms (MGFA Class II–V) increased following diagnosis and throughout the course of disease, suggesting generalization of the disease and an increase in disease severity over time. This describes the usual disease course and aligns with previous observations [4, 6, 19]. Despite 96% of patients being treated, the mean number of symptoms per patient overall was the same at diagnosis and at the time of survey completion, and the five most frequently reported symptoms experienced at time of survey completion were also similar to those reported at diagnosis. At any point over the course of disease, 45% of patients were classified with MGFA Class III or above, compared with approximately 27% at diagnosis, reflective of the known fluctuations in MG symptoms.

Many people with MG report a negative impact of disease on their life due to fatigue [20, 21] and muscle weakness, together with anxiety and depression [22–26]. In this study, 11% of patients had severe fatigue, and general fatigue was reported as the most “troublesome” symptom in all countries. These findings reflect how symptoms regarded as “troublesome”, such as fatigue and muscle weakness, are those that directly and negatively impact QoL and the ability to perform daily tasks [20, 24]. This is consistent with a study from Germany who report health-related quality of life is substantially lower in patients with MG compared with the general population, including more symptoms of fatigue, anxiety, and depression [27]. Patients with MG are known to be more likely to report anxiety and depression [28] and in our study, a large proportion of patients reported depression, anxiety, or both. Our study was conducted during the 2020 COVID-19 pandemic, during which rates of depression and anxiety increased from pre-pandemic years by about a quarter [29], so it is possible that the high rates of depression and anxiety reported here were influenced by the pandemic. Comorbidities add to the disease burden, from which two-thirds of patients in this survey were reported to suffer, and almost 86% of these patients received one or more concomitant medications. A quarter of patients with a comorbidity received antidepressants.

Our findings on treatment and symptom severity in MG demonstrated that despite chronic therapy, two-thirds of patients within this MG cohort were still moderate-to-severely symptomatic. Of those with ongoing moderate or severe symptoms, many patients were still being treated with first-line AChEI or steroid therapy. Patients who had previously required acute treatment were more likely to receive second- or third-line therapy. Data from the US medical claims database, IBM^®^ MarketScan^®^ Commercial Claims Encounters and Medicare supplemental, have also shown that many patients with MG experienced exacerbations and required rescue therapy despite treatment [30]. In the present study, over one-third of patients had previously received acute treatment, predominantly for the treatment of exacerbations or myasthenic crises, highlighting the lack of disease control with currently available maintenance treatments. Taken together, these data suggest there is a significant need for improved treatment options in MG to reduce the number of symptoms, disease severity and burden on patients.

Although there is some variability, HCRU and disease management were generally similar across all countries. As expected, neurologists were the most frequently involved HCPs overall and three-quarters of patients across countries were also seen in primary care, possibly reflective of a collaborative, multidisciplinary approach to management of patients with MG. Across countries, at least one-fifth of patients had been hospitalized in the last 12 months; the highest proportion of patients was seen in France, where one in three patients had been hospitalized. AChEIs, specifically pyridostigmine, were the most commonly prescribed treatment for chronic MG in all countries, reflecting international and UK guidelines that pyridostigmine should be used in the initial treatment in most patients with MG [31, 32]. Wide use of prednisone and azathioprine also reflected international treatment recommendations for adding corticosteroids to AChEIs or immunosuppressive treatments as second-line treatment, respectively [31]. However, despite international guidelines not reaching a consensus on the use of rituximab in MG, we found that rituximab was the fifth most commonly prescribed treatment overall [31].

The results from this study are supported by a growing body of real-world evidence from physician-reported questionnaires and claims databases that highlight how MG disease activity is not always adequately controlled [33–37], and that there is a need for improved treatment options and early treatment interventions. Many people with MG continue to have exacerbations and inadequately controlled disease despite maintenance treatment [38], and some still experience myasthenic crisis [39]. A real-world study conducted in Sweden reported that almost half of 1077 patients reported their symptoms as ‘dissatisfactory’ [35]. Approximately 10–20% of patients with MG receiving currently available treatments do not respond to treatment, which has a significant impact on health-related QoL [40, 41]. There are several, novel immunotherapy treatments currently in development for MG with promising efficacy and safety profiles compared with conventional treatments [42]. Complement inhibitors (eculizumab, ravulizumab, zilucoplan) and neonatal Fc receptor blockers (efgartigimod, rozanolixizumab, nipocalimab) are among the targeted treatments now available or in development that may help reduce the risks associated with broad-spectrum immunotherapies, and both short- and long-term steroid use [42, 43].

In addition to improved treatment options, patients also need an accurate and expedited diagnosis of MG. In the present study, on average, patients were waiting nearly 11 months for a diagnosis following their onset of symptoms. The Swedish study reported that patients may be waiting for up to 2.6 years for an MG diagnosis [35], during which time patients are left untreated and experiencing symptoms [44]. In the present study, in which MG diagnosis was based on the physician’s judgment, approximately a quarter of patients in this study were misdiagnosed on presentation of symptoms. Patients with MG were being misdiagnosed with nonspecific syndromes or psychogenic disorders, such as chronic fatigue syndrome or hysteria, highlighting the need for additional education in the medical community about MG and its symptoms and standardized procedures to establish a diagnosis of MG, so patients can be referred to specialists as soon as possible. Other real-world data from the US MarketScan^®^ database suggest that the early years following MG diagnosis are a period of particularly high healthcare burden, and therefore a rapid diagnosis is essential to avoid increased HCRU [45]. However, access to assessments by specialists varies between the medical systems used in different countries, with some systems providing direct access to specialist care and others exercising more control [46].

The limitations of this study are those inherent to real-world data collection and interpretation. Data capture was dependent on patients presenting to the physician in the fieldwork time frame, which generated a range of patients across treatment types and disease stages; however, there may have been a greater proportion of those patients who consulted or presented more frequently. While participants were required to complete each field in the form, “Don’t know” was a valid response option and so, as is often the case in survey-based methodologies, there are some missing data, particularly for historical data such as baseline ages at symptom onset and MG diagnosis. MGFA classification is used predominantly in clinical trials and not routinely in clinical practice, so the applicability of the disease severity data to clinical practice may be limited. Additionally, the study was conducted during the COVID-19 pandemic, which may have restricted face-to-face consultations, healthcare resource availability, and uptake of patient-reported forms, and the consulting patient population may have consisted of a higher proportion of patients with severe disease than would otherwise be expected during data collection. However, inclusion criteria for physicians and patients were minimal, which allowed broad, geographically diverse inclusion of consulting physicians and presenting patient populations.

## Conclusions

These physician- and patient-reported data confirm the need for education of non-neurologist specialists on identifying presenting signs and symptoms for a correct and timely MG diagnosis, and improved treatment options and diagnosis methods for patients with MG. Even when their MG is appropriately diagnosed in a timely manner, many patients with MG continue to experience symptoms and worsening of disease severity, and only a quarter of patients see an improvement following treatment. There remains a need for additional therapies to address the unmet need in patients with MG.

## Data Availability

All data, including methodology, materials, data and data analysis, that support the findings of this survey are the intellectual property of Adelphi Real World. All requests for access should be addressed directly to Jonathan DeCourcy (Jonathan.DeCourcy@adelphigroup.com).

## Abbreviations

ACE: Angiotensin-converting enzyme
AChEI: Acetylcholinesterase inhibitor
BMI: Body mass index
DSP: Disease Specific Programme
ER: Emergency room
EU: European Union
HCP: Healthcare professional
HCRU: Healthcare resource utilization
ICU: Intensive care unit
IVIg: Intravenous immunoglobulin
MG: Myasthenia gravis
MGFA: Myasthenia Gravis Foundation of America
MG-QoL-15r: 15-Item MG QoL
NMJ: Neuromuscular junction
PRQ: Physician-reported questionnaire
QoL: Quality of life
SCIg: Subcutaneous immunoglobulin
SD: Standard deviation
UK: United Kingdom
VAS: Visual Analog Scale
WPAI: Work Productivity and Activity Impairment
